# Dietary Management of Obesity: A Review of the Evidence

**DOI:** 10.3390/diagnostics11010024

**Published:** 2020-12-25

**Authors:** Nahla Hwalla, Zeinab Jaafar

**Affiliations:** Department of Nutrition and Food Sciences, Faculty of Agriculture and Food Sciences, American University of Beirut, P.O. Box 11-0236, Beirut 1107 2020, Lebanon; zeinabjaafar1294@gmail.com

**Keywords:** obesity, dietary management, weight loss, metabolic adaptation

## Abstract

Obesity is a multi-factorial disease and its prevention and management require knowledge of the complex interactions underlying it and adopting a whole system approach that addresses obesogenic environments within country specific contexts. The pathophysiology behind obesity involves a myriad of genetic, epigenetic, physiological, and macroenvironmental factors that drive food intake and appetite and increase the obesity risk for susceptible individuals. Metabolically, food intake and appetite are regulated via intricate processes and feedback systems between the brain, gastrointestinal system, adipose and endocrine tissues that aim to maintain body weight and energy homeostasis but are also responsive to environmental cues that may trigger overconsumption of food beyond homeostatic needs. Under restricted caloric intake conditions such as dieting, these processes elicit compensatory metabolic mechanisms that promote energy intake and weight regain, posing great challenges to diet adherence and weight loss attempts. To mitigate these responses and enhance diet adherence and weight loss, different dietary strategies have been suggested in the literature based on their differential effects on satiety and metabolism. In this review article, we offer an overview of the literature on obesity and its underlying pathological mechanisms, and we present an evidence based comparative analysis of the effects of different popular dietary strategies on weight loss, metabolic responses and diet adherence in obesity.

## 1. Obesity: Prevalence and Magnitude

**Figure 1:**
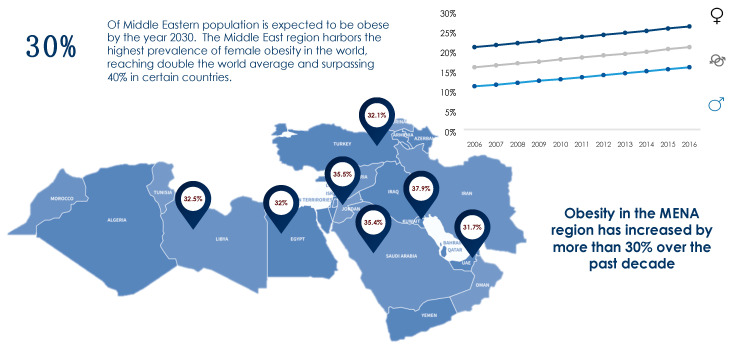
Prevalence of obesity in the MENA region based on WHO (2016) records [2].

**Figure 2:**
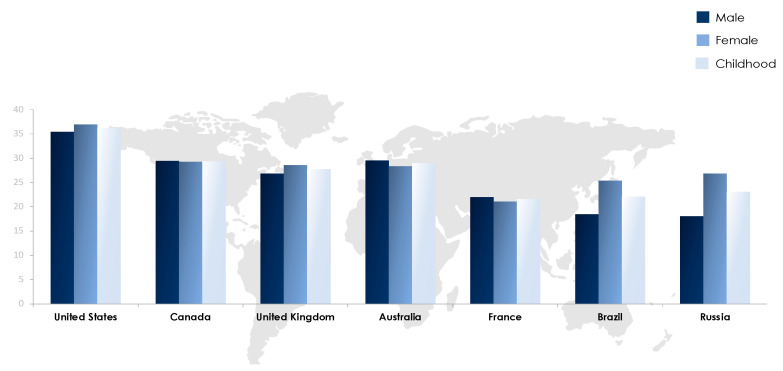
Prevalence of obesity in selected countries worldwide based on WHO (2016) records [2].

The obesity epidemic is one of the most predominant public health challenges of the century and its prevalence continues to rise globally with now more than 500 billion obese individuals around the world [1]. Being associated with over 60 comorbid conditions and at least 12 different types of cancers, obesity poses significant health and economic burdens on affected individuals as well as on the sustainability of public health systems in all parts of the world, but more profoundly in developing countries. The figures of obesity in the Middle East are staggering, particularly in the Gulf region, and the prevalence of female obesity surpasses 40% in some countries such as Egypt, Jordan and Saudi Arabia [2] (Figure 1). Over the past decade, the prevalence of obesity in the Middle Eastern and North African (MENA) region has increased by more than 30% and has been accompanied by a concomitant surge in the prevalence of non-communicable diseases (NCDs). For example, the prevalence of type 2 diabetes, which is strongly related to that of obesity, was estimated to surpass 15% of the adult populations in certain countries of the MENA region such as Egypt (15.2%), Bahrain (16.3%), Qatar (15.5%), Saudi Arabia (18.3%), and the United Arab Emirates (15.4%) [3]. Moreover, according to the International Diabetes Federation (IDF), the MENA region had the highest world-age standardized prevalence of diabetes globally in 2019 at 12.9% (95% confidence interval 7.2–17.6%) [3]. The dramatic and exponential rises in prevalences of obesity, diabetes and other NCDs call for immediate interventions addressing these serious epidemics. Data on the prevalence of obesity in different countries of the MENA region and the world from World Health Organization (WHO) 2016 data records are summarized in Figure 1 and Figure 2.

## 2. Population-Wide Initiatives to Combat Obesity

Obesity has been recognized as a major challenge for healthcare systems and economies around the world and has emerged on the public health agendas of numerous countries and international organizations which have set in place policies and strategies to enable individuals and societies to change their behaviors and mitigate obesity prevalence and risks. These policies range from targeting individual behaviors such as mass media campaigns to managing the food and built environment such as fiscal measures and regulation of food advertisements and labelling, and a convincing body of evidence corroborates their cost-effectiveness [4,5]. Examples of different types of population-wide interventions to combat obesity are summarized in Table 1.

In a microsimulation modelling and systemic review study by Gortmaker et al. the implementation of a sugary beverages tax in the United States has been simulated to prevent 575,936 childhood obesity cases over a period of 10 years and save around 14 billion United States Dollars (USD) in net costs, primarily due to reductions in adult health care costs [5]. Gortmaker et al. also evaluated the cost-effectiveness of 6 other interventions (restaurant menu labelling, elimination of tax subsidy on advertisement of unhealthy foods for children, nutrition standards for school meals, nutrition standards for foods and beverages sold in schools, improved early care and education policies and practices and increased access to adolescent bariatric surgery) and concluded that even though no single intervention is capable of producing significant reversal of obesity on its own, macro-level solutions to obesity could cumulatively bring about positive health and economic outcomes to societies and healthcare systems [5]. The adoption of the Mediterranean and Dietary Approach to Stop Hypertension (DASH) diets has also been suggested as a population wide approach against obesity due to their well-recognized benefits to health and weight loss, their socio-cultural feasibility and generalizability to different populations [6].

Case studies from different countries that have set national strategies to combat obesity provide realistic evidence on the success of population wide interventions, beyond modelling and research-controlled settings. Though multiple studies demonstrate successes of such interventions, a systematic review by Sisnowski et al. [7] evaluating 6 types of public health approaches (menu labelling, product labelling, subsidies on healthy foods, taxation of unhealthy foods/beverages, procurement standards for public institutions and improvement of food infrastructures) based on evidence from different parts of the world, indicated that the current policies are generally failing to achieve significant effects on consumers behaviors to a degree that would impact the incidence of overweight, obesity or associated NCDs. Moreover, the minimal effects on individuals’ behaviors seem to disappear over time. Sisnowski et al. highlighted the fact that most anti-obesity public health interventions do not match the advocated recommendations found in the literature, possibly due to industry opposition, commercial profit pressure and lack of political will [7]. The case of Mexico provides an example on the successful implementation of anti-obesity interventions that matches the recommendations of public health experts [7]. In 2014, Mexico introduced a 10% tax on sugar-sweetened beverages and an 8% tax on non-essential energy dense foods due to the alarming rates of obesity and NCDs in the country. Their plan also included recommendations on healthy hydration, restrictions on the advertisement of unhealthy products and product labelling, and strategies targeting schools and the workplace among others [8]. The success of the Mexican example has been highlighted on many occasions [9,10,11] including a study by Colchero et al. which showed a decline of 7.6% in purchases of taxed beverages after 2 years of implementing the plan, though effects on energy intake and diet quality remain unstudied [9]. Whilst it is still too early to evaluate the influence of such measures on population health and obesity, the observed decrease in purchase of sugary drinks infers positive outcomes [12]. The Mexican experience has encouraged many countries to adopt similar fiscal measures such as Chile, Barbados, the United States and the United Kingdom [13,14,15]. Arab countries including Qatar, UAE, Saudi Arabia have also demonstrated efforts to combat obesity at the governmental level including policies for menu labelling and taxations however, these efforts require more institutionalization and focus to ensure proper implementation and sustainability [16]. While current population wide approaches to tackle obesity may not offer immediate impact on health outcomes, several factors should be considered including long term effects and whether their magnitude matches what is recommended.

## 3. The Interplay of External and Internal Culprits

Historically, human obesity was commonly associated with gluttony and lack of self-control and as such treatments were primarily directed towards individual behaviors [17]. However, research over the past decades has identified a myriad of genetic, epigenetic, physiological and environmental factors that interact within a highly convoluted framework to influence food intake and eating behaviors, leading to overweight and obesity.

### 3.1. Role of Gene-Environment Interaction

The dramatic rise of obesity over the past decades has emphasized the detrimental influence of the food and built environments on people’s food choices and activity levels. The past decades have witnessed systematic shifts in people’s dietary patterns, consumption behaviors and physical activity levels around the world that were concomitant to economic growth, globalization of trade and food systems, mechanization and mass urbanization. These global changes are amongst the main contributors that have facilitated the creation of an obesogenic environment that promotes overeating and sedentariarism, thus favoring a positive energy balance and “passive” weight gain [17,18,19]. On a macro-level as well, governmental policies and economic systems regulating different food and beverage-related industries including food production, transport and marketing contribute significantly to the abundant availability, easy accessibility, affordability, and thus overconsumption of unhealthy food beyond the personal needs. A considerable body of evidence highlights the detrimental impacts of policy decisions on the self-regulation of food intake and appetite, and consequently on the maintenance of body weight, especially in children where personal responsibility is relatively limited [20,21].

The environmental influence on obesity-related gene expression has also been widely studied. The hereditary nature of this disease as well as the gene-environment interactions explain the wide variation that exists in individuals’ responses to similar environmental and internal cues, and their susceptibility to develop obesity. The role of heredity in the etiology of obesity is estimated to be in range of 40–70%, and while genome wide association studies (GWAS) have identified more than 800 genetic loci associated with obesity, most BMI variability remains unexplained [22,23]. The missing heritability is thought to be partly covered by epigenetic interactions whereby a number of environmental factors such as dietary intake, toxins and pollutants, may induce epigenetic changes such as DNA methylation, histone modifications and non-coding RNAs, that can remain stable and passed on to future generations, further highlighting the complexity of the interplay between external and internal determinants of obesity [24]. In a recent study by Sulc et al. (2020), gene-environment interactions have been estimated to account for 1.9% of variance in 9 obesity-related traits (e.g., trunk fat free mass), though further investigations are required before having any impact on public health interventions [25]. Many studies have investigated the epigenetic effects of specific nutrients and substances of dietary origins. For instance, butyric acid, majorly found in cheeses, has been found to inhibit histone deacetylase thus possibly acting as an antitumor agent [26]. In another study, palmitate affected DNA methylation in 73 BMI-related genes as well as genes associated with type 2 diabetes and dysregulation in fatty acid metabolism [27,28].

### 3.2. Regulation of Food Intake—Homeostatic and Hedonic Systems

**Figure 3:**
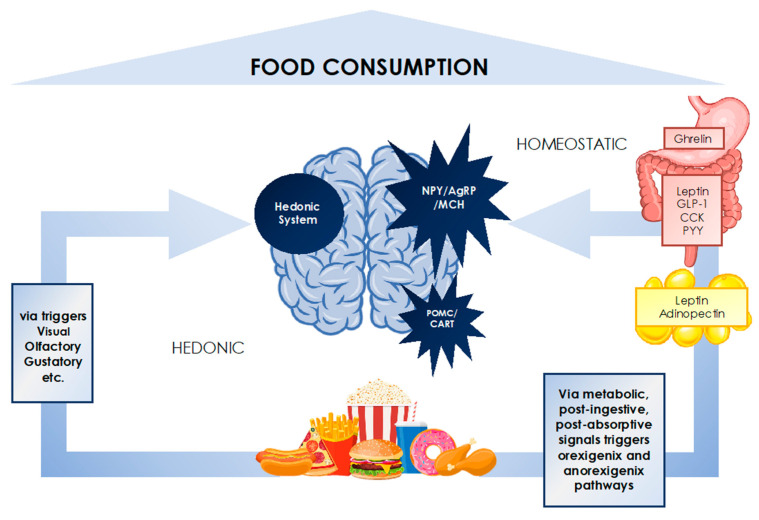
The interplay of homeostatic and hedonic systems in food consumption regulation.

The human body is equipped with a complex regulatory system that, based on a set of cues including sensory, physiological, metabolic, post-ingestive, post-absorptive and psychosocial signals, modulates the sensations of appetite, hunger, satiation and satiety, and as such food intake (Figure 3) [29,30].

This system involves a series of neuroendocrine cascades between the central nervous system, the gastrointestinal tract, the adipose tissue and other endocrine tissues, that adjust metabolic responses in the aim of maintaining energy and body weight homeostasis, similar to other physiological parameters like body temperature or blood pressure [30]. Under conditions of restricted caloric intake such as dieting, this homeostatic circuitry elicits compensatory mechanisms that increase appetite, hunger and cravings, decrease metabolic rate, mitochondrial efficiency and energy expenditure, and promote energy intake, posing great challenges to diet adherence and ultimately leading to weight regain on the long run [31,32]. Researchers have uncovered a wealth of knowledge on the molecular mediators of this intricate process involving gut hormones, neuropeptides, neurotransmitters, adiposity signals and endocrine markers. Nevertheless, the definitive roles and mechanisms behind each of these molecules are not fully understood and are yet to be uncovered [33].

The hormone ghrelin remains the only orexigenic hormone identified to date; ghrelin is produced in the gut and acts via the central nervous system (CNS) to trigger increased hunger and meal initiation and is reduced following food intake. The degree of ghrelin suppression is thought to be dependent on the type of ingested nutrients, as carbohydrates and proteins have been shown to decrease ghrelin more potently than lipids [33]. Among the anorexigenic mediators, cholecystokinin (CCK), peptide YY (PYY), glucagon like peptide-1 (GLP-1) and leptin have been most prominently studied. CCK is considered to be the primary gut hormone involved in appetite regulation and is secreted in the duodenum and released by certain neurons in the brain. CCK acts via receptors in the CNS to induce satiety, but also at level of the gut by promoting gastrointestinal motility and delaying gastric emptying. PYY and GLP-1 are secreted alongside each other by different parts of the gastrointestinal (GI) tract and induce satiety via direct central effects but also by delaying gastric emptying. GLP-1 also functions as an incretin hormone, acting on pancreatic β-cells to stimulate the secretion of insulin and inhibit that of glucagon, thus decreasing food intake [33,34].

Among adiposity signals, the role of leptin has received the greatest attention due to its pivotal role in the long-term regulation of food intake, energy balance and thus body weight. Leptin is primarily released by the adipose tissue and in proportion to its mass, and acts on several regions in the brain and the hypothalamus, stimulating the expression of anorexigenic signals (POMC, CART and corticotrophin releasing hormone), and inhibiting orexigenic pathways (NPY, AgRP, MCH). In the obese, the hypothalamic effect of leptin is diminished by leptin resistance [30]. During weight loss, leptin levels decrease quickly which induces powerful hunger signals and promotes food intake and weight regain [21].

Beyond the homeostatic control, there exists a different set of signals known as the hedonic system which, in response to the reward value of the food, stimulate pleasure-encoding regions of the brain that in turn increase the drive to eat, independently of the physiological needs [35]. There is increasing evidence on the ability of the hedonic system to override homeostatic control, promoting non-homeostatic overconsumption of food [30]. Key elements of the hedonic pathways are found in the cortico-limbic regions of the brain [36]. In appetite research, a greater activation of these regions on functional magnetic resonance imaging (fMRI) in response to food images has been reported to increase satiety and food consumption [37,38] and affect short-term weight loss or weight gain [39,40].

The mechanisms behind the regulation of food intake and appetite implicated in the etiology of obesity are highly complex and include multiple other modulators and pathways that have not been discussed in this paper. The interaction of homeostatic and hedonic systems is highly complex and determines individuals’ eating behaviors and cognitive choices of what and when to eat. These interactions fall behind the relatively recent epidemic of obesity whereby the ubiquity of marketing and availability of cheap, palatable and energy dense foods overwhelm the cognitive controls and overpower the homeostatic regulation of food intake leading to overeating and obesity. In the obese, attempts to lose weight need to focus on all these factors [41].

## 4. Dietary Interventions in the Management of Obesity

Understanding the biology behind weight regulation and food intake is extremely relevant in the management of obesity, and a key requisite to the design of effective weight loss strategies. The manipulation of dietary intake, in the aim of influencing obesity outcomes, has been the cornerstone of obesity management for centuries. One of the central tenets of weight loss and obesity prevention strategies is the restriction of energy intake. Energy restricted diets are commonly prescribed by dietitians and healthcare professionals as a first line treatment for obesity and recommended by most scientific societies and dietary guidelines. Generally, these diets follow the golden rule that decreasing daily energy intake by around 500 calories would translate into a weight loss of around 0.5 kg per week and around 2 kg per month [42].This is often achieved by controlling portion size, reducing intake of carbohydrates, total fat and saturated fat and increasing intake of proteins and fiber from fruits and vegetables, all aiming at decreasing the diet’s overall energy density and enhancing its satiating effect [43]. Despite the general agreement on the scientific soundness behind these approaches, the evidence from long term studies indicate a modest effectiveness at best, a highly heterogenous response, and a low maintenance of the lost weight [44]. In a meta-analysis of 29 long term trials on weight maintenance, only a 3% weight loss was maintained after a period of 5 years [45]. The extremely high rate of failure to maintain lost weight, estimated to be at more than 80%, is thought to be primarily the result of the metabolic adaptation and compensatory mechanisms that defend body weight and maintain energy stores [41,46].

The metabolic responses to energy restriction and weight loss have been extensively studied yet some parts of it remain elusive. An upregulation in levels of hunger hormones (ghrelin) and cortisol, significant reductions in anorexigenic hormones (leptin, insulin, GLP-1, PYY, CCK), testosterone, thyroid hormones and energy expenditure (around 28% reduction), and even heightened perception of reward value of foods are amongst the compensatory mechanisms that follow weight loss, promoting energy intake and leaving dieters vulnerable to weight relapse [31]. This alteration in endocrine profile has been found to persist beyond the period of weight loss, for one year at least.

Different nutritional approaches and strategies have been proposed to influence the feedback system outlined earlier, exploiting its sensitivity to the composition of the diet in different ways and on multiple fronts. These approaches can be broadly categorized into macronutrient-focused (e.g., low carbohydrate, low fat, high protein diet) which highlight the differential contribution of macronutrients to metabolism and energy homeostasis, dietary pattern focused (e.g., Mediterranean diet) and dietary timing focused (e.g., intermittent fasting) [47]. In the sections below, we summarize the most recent evidence on the effectiveness of five dietary approaches used popularly in the management of obesity (high protein, low carbohydrate, low fat, Mediterranean diet and intermittent fasting) with the main outcomes as weight loss and adherence. We also highlight the main metabolic advantages and disadvantages that underlie each of these dietary approaches in achieving weight loss and maintenance.

### 4.1. Macronutrient-Focused Approach

#### 4.1.1. Effects on Weight Loss

The modification of a diet’s macronutrient composition has been the foundation for many individual weight loss strategies aiming to overcome the metabolic compensation and poor adherence associated with energy restricted diets [48]. The debate on the superiority of one macronutrient composition over the other in achieving weight loss has long historical roots and remains unsettled. To date, the evidence does not support any particular macronutrient balance over another, and differences in weight loss have only been observed when accompanied by a significant energy deficit (Table 2). 

In one of largest trials examining the effects of different macronutrient profiles, the Preventing Overweight Using Novel Dietary Strategies (POUNDS lost) study, similar weight loss and body composition changes were observed at 6 months and 2 years [59]. These findings have also been corroborated by a number of meta-analyses and systematic reviews of randomized clinical trials (RCTs), indicating that different macronutrient combinations can lead to similar weight loss outcomes with minimal differences in weight loss on the short term (a mean difference of less <1 kg at 6 months or less) as long as there is good adherence to the diet (Table 2). Most recently, Ge et al. have published a meta-analysis of 121 RCTs comparing the effectiveness of 14 popular diets including macronutrient-focused diets and dietary pattern focused diets (Mediterranean diet and DASH), with the main outcome being degree of weight loss in addition to changes in cardiovascular markers (LDL, HDL, blood pressure, C-reactive protein) at 6 and 12 months follow ups [58]. Based on the findings, low fat and low carbohydrate diet were found to achieve greater weight loss compared to usual/habitual diets but were similar to each other. Weight reduction was noted to a lesser degree with Mediterranean diet and other macronutrient balanced dietary patterns, however, at 12 months, all effects on weight loss and CVD markers were negligible for all diets [50]. Beyond the effect on weight loss, recent decades have brought a greater understanding of the differential effect of macronutrients on the metabolic regulation of food intake and appetite. While it might not necessarily influence weight loss outcomes, the manipulation of macronutrient content could bring about metabolic benefits and alter food intake in a way that helps dieters enhance their adherence to the diet [60,61].

#### 4.1.2. Effect of Metabolic Regulation and Adherence

##### High Protein Diets

Studies on both obese and non-obese individuals report high protein diets to have a greater satiating effect and to better preserve fat free mass following weight loss compared to other iso-energetic macronutrient focused diets, which could help maintaining a negative energy balance [50,62,63,64]. The strong satiating effects of high protein diets are thought to result from the combined expression of several mechanisms triggered either directly or indirectly by the increased levels of circulating amino acids. Evidence consistently report an increase in levels of anorexigenic hormones (GLP-1, PYY, CCK) or a greater decrease in levels of orexigenic hormones (ghrelin) and low levels of leptin following a high protein intake, which in turn would act via the central nervous system to suppress food intake [65,66,67,68,69,70]. Animal studies showed the altered metabolic profile to persist for 12 weeks in mice ingesting a high protein diet [20,71], however this requires further investigations in humans for longer periods of time. Another classical mechanism behind protein’s satiating effect is the increased diet-induced thermogenesis (DIT) which contributes to increased energy expenditure. Studies have shown that dietary protein significantly increases DIT, sleeping metabolic rate and basal metabolic rate in a 36-h respiration chamber compared to isocaloric dietary carbohydrate and fat of the same volume [67,72]. One important reason underlying protein’s higher thermic effect as compared to other macronutrients is that it necessitates immediate metabolic processing due to the body’s inability to store it, in addition to the higher ATP cost of peptide bond synthesis and urea production [62,63]. The generally poor palatability of high protein diets has also been implicated in its food intake suppressing effect [63]. A high protein intake has also been suggested to induce hepatic gluconeogenesis in rodents [63]. By modulating glucose signaling in the brain, gluconeogenesis could be an additional mechanism by which high protein intake may induce satiety. However, this theory has not been well-supported by evidence, and a study by Azzout-Marniche et al. demonstrated that a high protein diet without any carbohydrate intake did not stimulate gluconeogenesis sufficiently to signal satiety [73].

The metabolic and appetite suppressing effects of proteins are thought to be dependent on the type and quality of proteins which are determined by the amino acid composition (essential vs. non-essential; ketogenic vs. glucogenic) [63]. On the longer term, satiety induced by dietary protein is not thought to be sustained as the human body seems to adapt to the high protein intake by increasing protein turnover and amino acid oxidation [62]. With regards to the effect on weight loss, RCTs conducted on obese individuals generally indicate comparable effects between high protein diets and other iso-caloric diets of normal protein intake that are either lower in fat or carbohydrates, however some studies report inconsistent findings [74,75,76,77]. The effects of high protein diets coupled with different carbohydrate and fat compositions have also been examined in the literature. In a RCT by Johnstone et al. high protein low carbohydrate diet (4% carbohydrate) and a high protein moderate carbohydrate diet (35% carbohydrate) had comparable effects in improving markers of insulin resistance, lipaemia and inflammation in obese individuals [78]. Though high protein low carbohydrate diet led to greater weight loss, this was attributed to larger loss in total body water. An earlier RCT by Hwalla et al. had shown a high protein diet (45% protein, 25% carbohydrates, 30% fat) to induce greater weight loss, a lower decrease in resting energy expenditure and normal levels of mean fasting insulin in hyper-insulinemic obese subjects compared to a high carbohydrate diet (12% protein, 58% carbohydrates, 30% fat) which was also shown to decrease insulin levels but not to the extent of normalization [79].

On the other hand, a RCT by Noakes et al. found that a high protein diet coupled with low fat intake to achieve better nutritional and metabolic outcomes in overweight and obese women compared to a high carbohydrate diet [76]. A greater weight loss following the high protein low fat diet was only observed in women with elevated plasma triglycerides, whereas no difference was observed in women with low triglycerides levels. This study was amongst the first to highlight a possible phenotype-diet interaction which requires confirmation from other studies.

##### Low Carbohydrate Diets

Similar to high protein diets, low carbohydrate diets have gained wide popularity due to their ability to suppress food intake and appetite, induce fat oxidation and rapid weight loss, and improve metabolic markers, primarily via ketogenesis. The drop of insulin levels that follows a low carbohydrate intake reverses the inhibition of the hormone lipase and increases the breakdown of fat from adipose tissue and the formation of ketone bodies in the liver. With the insufficient levels of glucose in the body, ketones become the primary source of energy in the body. The anorexigenic effects of ketone bodies on satiety and hunger have been widely reported in the literature including a meta-analysis by Gibson et al. where ketogenic diets have been shown to prevent a rise in appetite following weight loss, and helped overweight and obese dieters feel less hungry at the fasting state and fuller after eating [80]. A higher plasma concentration of ketone bodies following dietary induced ketosis is associated with decreased secretion of ghrelin and lower subjective hunger [81]. According to Paoli et al. ketone bodies, namely *β*-hydroxybutyrate, decrease the expression of NPY and AgRP at the level of the hypothalamus, increase levels of CCK and decrease that of ghrelin after meals [82]. The use of exogenous ketones such as ketone esters for weight loss purposes is also an emerging field but requires more extensive research to evaluate its safety and efficacy [83]. The role of ketone bodies in the hunger-satiety cycle is however, contradictory as they can exert both orexigenic and anorexigenic effects. In the orexigenic pathway, ketone bodies have been found to increase the levels of adiponectin, *γ*-aminobutyric acid (GABA) and AMP-activated protein kinase phosphorylation, and decrease the production of reactive oxygen species (ROS) at the level of the brain, which would increase food intake and decrease satiety [82]. Hypothalamic ROS are thought to modulate energy homeostasis, promote satiety, and to be essential for the eating-suppressive effects of insulin [82,84]. Another mechanism by which low carbohydrate diets might induce rapid weight loss is diuresis as a consequence of glycogen depletion since 1 g of glycogen is associated with 3 g of water [84]. Some evidence supports the effects of the higher protein intake rather than the lower carbohydrate intake in inducing weight loss, primarily via proteins’ satiating effects and increased thermogenic effects, in addition to the poor palatability of the diet [85].

To make up for the reduction in carbohydrate, intake of fat is often increased. The question around the safety of low carbohydrate high fat diets continues to be a subject of controversy with limited evidence examining their long-term effects. In a RCT by Retterstol et al., a low carbohydrate diet coupled with high dietary fat intake has been found to increase plasma LDL-C by 44% (range between 5% and 107%) compared to the control group in normal weight individuals over a period of 3 weeks [86], which could increase CVD risk. However, studies on the metabolic effects of low carbohydrate high fat diets, namely on lipid profile, report inconsistent findings and while some do not note any differential impact, others indicate improvements in lipid markers among a range of other metabolic benefits including greater reductions in HbA1C and better glycemic control in patients with type 2 diabetes [87,88,89]. A low intake of carbohydrates, namely dietary fibers, is also known to be associated with negative effects on the gut function and overall health [90,91]. Brinkworth et al. associated a low carbohydrate energy restricted diet with adverse impacts on bowel activity in overweight and obese individuals related to the low fiber intake and decreased production of short chain fatty acids by the microbiome in the large intestine, which may potentially contribute to the development of bowel disease on the longer run [91]. In a study by Kennedy et al. examining the association of food quality in different diets (measured by the food index score) and health effects, low carbohydrate diets (<30%) were assigned the lowest score and thus lowest diet quality [92].

The evidence on the effectiveness of low carbohydrate diets in weight loss is also highly inconsistent primarily due to high variability in the quantity of ingested carbohydrates and the durations of the studies; while a number of meta-analyses indicate an advantage of low carbohydrate diets over low fat and balanced diets [56,61,93,94,95,96], others show similar weight loss outcomes [57,97,98,99] as shown earlier in Table 2. While low carbohydrate diets might be effective for short term weight loss, compliance on the long term is both difficult and potentially dangerous since a significant reduction of carbohydrate intake in addition to a high fat intake may lead to increased low-density lipoprotein (LDL) cholesterol levels and increase mortality risk [42,87,100].

##### Low Fat Diets

An alternative macronutrient focused approach is the low-fat diet. The relationship between fat intake and body weight has been studied for decades and low-fat diets have been historically recommended for weight loss based on several observations: fat is the most energy dense and least satiating macronutrient and decreasing its intake would significantly reduce caloric intake [42]. These recommendations also take into account the significant correlation between fat intake, mainly saturated and trans fats, plasma cholesterol and cardiovascular disease (CVD) risk. Despite the theoretical advantage, meta-analyses and RCTs have failed to prove that low-fat diet can lead to a greater weight loss than iso-caloric counterparts. In a systematic review of 53 studies including normal weight, overweight and obese individuals, low-fat diets were found to be equally or even less effective in maintaining long term weight loss compared to interventions of higher fat content and of similar intensity [59]. In 2 meta-analyses of RCTs, combining low-fat with a higher protein intake produced more favorable outcomes for weight loss, satiety, plasma triglycerides, resting energy expenditure and fat mass compared to low-fat standard protein or low-fat high carbohydrate diets [54,101]. The outcomes of combining a low fat with a high carbohydrate intake may be however less promising. In a study by Tessitore et al. [102], mice fed a long-term low-fat high carbohydrate diet exhibited hepatic damage which was accompanied by significant increase in levels of hepatic inflammatory mediators (interleukin-1β, interleukin-6, tumor necrosis factor-α and hepatocyte growth factor) and was comparable to mice fed a high fat diet and not observed in mice fed a standard diet. After 18 months, 30% of mice eating a low-fat high carbohydrate diet showed nodules with defined neoplastic features. The observed hepatic damage has been hypothesized to be a result of the increased carbohydrate intake, namely fructose. Fructose is known to be rarely adsorbed by the gut and almost entirely cleared by the liver. In the liver, phosphorylation of fructose produces metabolites that can cause hepatic fat accumulation which consequently leads to damage and tumorigenesis [98].

From a satiety standpoint, a 12 months RCT by Hu et al. found low-fat diet was found to reduce PYY and thus satiety more than a low carbohydrate diet in obese individuals while there was no effect on ghrelin nor on self-reported appetite in either groups [103]. This study additionally reported the changes in PYY to be 99% related to the macronutrient composition and not to weight loss [100]. These findings however, have been contradicted in other studies (of smaller sample size) that indicated no difference in effects on PYY between low carbohydrates and low-fat diets, also in obese individuals [104,105]. Moreover, there is considerable evidence from two large and long-term studies (Women’s Health Initiative and Look AHEAD studies) that the beneficial impact of low-fat diets on CVD is marginal at best, whether accompanied with restricted energy intake or not [106,107].

### 4.2. Dietary Pattern-Focused Approach (The Mediterranean Diet)

The Mediterranean dietary pattern has been widely recognized for its beneficial health effects, and data from several RCTs, namely the Prevención con Dieta Mediterránea (PREDIMED) study, highlight its protective role against cardiovascular diseases, type 2 diabetes mellitus, breast cancer and age-related cognitive dysfunction among other disorders, in addition to decreasing all-cause mortality [108,109,110,111]. The main mechanisms underlying the diet’s beneficial effects are not fully uncovered but are thought to include decreasing plasma lipid levels, alteration of hormones and growth factors implicated in carcinogenesis, protection against oxidative stress and modulation of gut microbiota composition [109]. The Mediterranean diet has also been implicated in obesity prevention and treatment, primarily due to its low energy density and high fiber content, a notion that has been subject to conflicting views hypothesizing that the high fat Mediterranean dietary pattern involving the intake of unrestricted amounts of olive oil and nuts would promote weight gain and adiposity. The PREDIMED study provides first level evidence that the long-term adherence to the Mediterranean diet results in little to no change in body weight and adiposity. Compared to other dietary approaches, a meta-analysis by Mancini et al. showed that the Mediterranean diet results in greater weight loss than a low-fat diet after a minimum period of 12 months, but leads to similar outcomes when compared to a low carbohydrate diet and the American Diabetes Association Diet [112]. Furthermore, data from RCTs show that replacing high glycemic index foods with minimally processed plant-based food, typical of the Mediterranean diet, without a reduction in calories, contributes significantly to weight loss [112]. A satiating effect has also been attributed to metabolism of resistant starch and oligosaccharides by the gut microbiome, producing short chain fatty acids and satiety hormones and decreasing gastric emptying [113]. Animal studies also report that SCFA, namely propionate and butyrate, have an essential role in the prevention of obesity-related insulin resistance at the level of the brain, by activating the Free Fatty Acids Receptors 2 and 3 (FFAR2 and FFAR3) [114,115,116] which suppresses the expression of orexigenic neuron PYY [117] and attenuates ghrelin receptor signaling thus leading to appetite control [118]. Studies in rodents additionally show that the administration of prebiotics induces changes in the gut microbiome composition that favors the production of butyrate, which is associated with increased levels of GLP-1 [119]. In humans, the infusion of SCFA in the rectum of overweight individuals was associated with an increase in the levels of PYY [120].

### 4.3. Dietary Timing-Focused Approach (Intermittent Fasting)

Intermittent fasting has recently received widespread popularity in the management of obesity both in the scientific community and the media. It generally involves the partial or complete restriction of energy intake for pre-determined periods of time (e.g., time-restricted fasting, alternate day fasting) while allowing ad libitum intake at all other times [121]. The basic premise of intermittent fasting is the alteration of hormonal secretions and metabolic pathways leading to improvements in metabolic markers such as insulin sensitivity, blood pressure, blood glucose, lipid profile and inflammatory mediators among others [122]. The mechanisms linking fasting to metabolic changes are thought to include effects on circadian rhythm, alteration in the composition and activity of gut microbiome, and shifts in lifestyle behaviors (e.g., sleep) [123]. In animals, intermittent fasting has been associated with positive outcomes for diseases such as cancer, diabetes and CVD [122]. In humans, a fasting period of 12–24 h has been associated with a 20% reduction in blood glucose and glycogen store in the liver, leading to a ketogenic state and the usage of the body’s fat stores for energy [123]. Based on the available evidence, it seems that any form of intermittent fasting can lead to some degree of weight loss and positively impact metabolic markers namely insulin resistance and cardiovascular indicators without significant side effects. The level of weight loss reported in overweight and obese individuals following intermittent fasting diet has been averaged at 4–10% over a period of 4–24 weeks [124]. Nevertheless, the evidence stems largely from observational and cross-sectional studies of small sample size with little to no examination of the long-term effectiveness and safety of this dietary approach. It is also important to highlight that fasting diets might not be suitable for all age groups including children and older persons as they might be associated with side effects such as fatigue and weakness [124]. While intermittent fasting may lead to short-term weight loss, it is unlikely that its underlying mechanism is beyond the achievement of energy deficit and recommending it for long term use requires higher quality evidence. With regards to its metabolic benefits and positive effects on disease outcomes, the current evidence is promising and provides a strong rationale for larger trials.

**Figure 4:**
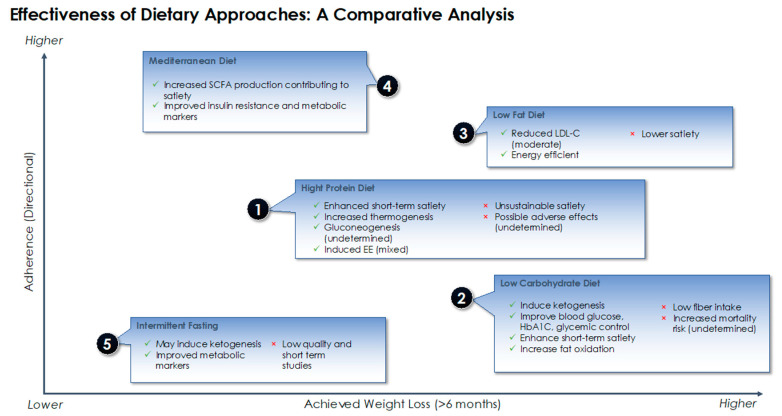
Comparative analysis of different dietary approaches in terms of weight loss, adherence and metabolic effects.

In Figure 4, we present a comparative analysis of the different dietary approaches discussed in this paper (Figure 4) based on their effectiveness in weight loss and degree of adherence. While just about any calorie restrictive diet can achieve a certain degree of weight loss, long term adherence is often a challenge. Dietary adherence is an important empirical indicator of the success of dietary interventions and is influenced by a number of factors including personal preferences and motivation but also the diet’s metabolic effects and the degree of food restriction [124]. Based on our interpretation of the evidence, a Mediterranean dietary pattern which invokes having a balanced, less restrictive intake and making small improvements to one’s diet ranks higher in terms of long-term adherence compared to other diets included in this review. The Mediterranean diet’s effectiveness in weight loss and health advantages are also supported by a wealth of evidence, and as this diet has not been correlated with “harmful” metabolic alterations, it has been ranked as most the effective approach for weight loss. Among macronutrient focused approaches, a low carbohydrate diet was found to generally achieve better weight loss outcomes, though comparable to diets low in fat. However, some evidence associates low carbohydrate diets with negative health and metabolic influences related to the long-term effect of ketogenesis, leading to lower adherence on the longer term. Due to generally lower palatability and possible adverse effects, high protein diets were ranked lower than low fat diets in terms of adherence. As for intermittent fasting, more evidence is required to assess its effectiveness in weight loss and long-term adherence.

## 5. The Epigenetic Effects of Diet

A plethora of studies have related different nutrients, dietary patterns, and lifestyle choices to epigenetic changes that may have a pathogenic role in the development of diseases including obesity. The first 1000 days of life from conception represent a window of epigenetic plasticity whereby environmental cues such as maternal malnutrition or high fat diet could modulate fetal growth, organ maturation and susceptibility to disease [125]. There exist historical records linking poor maternal nutrition to the development of diseases and metabolic derangements such as diabetes, CVD and cognitive disorders in both the offspring and even the grand-offspring. The most prominently known evidence on the transgenerational heritability of epigenetic changes is the Dutch famine in 1944 whereby starvation in one generation led to poor health outcomes in subsequent generations. The DNA isolated from these individuals presented increased expression of insulin growth factor 2 (IGF2) and below-average methylation of its gene, among other epigenetic modifications, that persisted for six decades later [126]. These epigenetic changes were associated with a number of metabolic disorders including elevated total cholesterol, triglycerides, LDL-C, LDL-C to high-density lipoprotein cholesterol (HDL-C) ratio, and lower levels of HDL-C [127]. The fetal origins of adulthood disease have in fact been linked to both maternal and paternal diets and the linking mechanisms are suggested to include the methylation of gametes, mitochondrial dysfunction and oxidative stress in response to a restricted nutritional intake. Other nutritional factors known to have epigenetic effects are summarized in Table 3.

Even though perinatal period contributes most significantly to phenotypic plasticity, evidence suggests that dietary factors after birth and even during adulthood may also lead to epigenetic changes. For instance, in a study by Yuan et al. [123], lactation in rodents has been shown to cause epigenetic changes that are linked to adult obesity. Milk lipids activate the nuclear receptor PPARα (peroxisome proliferator-activated receptor) which is known to regulate transcription at the level of the liver. PPARα has been shown to induce PPARα dependent demethylation of Fgf21 (fibroblast growth factor 21) which is a liver hormone involved in a multitude of metabolic roles including body weight and energy homeostasis. The study correlates the demethylation of Fgf21 with a decreased risk of diet-induced obesity in older rodents thus highlighting a link between breastfeeding and suppression of obesity [28]. Additionally, in a study by Madkour et al. intermittent fasting during Ramadan has been shown to improve the expression of anti-oxidant and anti-inflammatory genes (*TFAM*, *SOD2*, and *Nrf2*) and thus may have a protective role against oxidative stress which is a primary etiological factor in multiple metabolic disorders including obesity [132].

## 6. The Way Forward

Despite the great body of research, the evidence comparing different dietary approaches in the management of obesity is challenged by a number of factors namely limited follow up data on long term effectiveness and adherence to dietary interventions which constitutes a huge gap in knowledge on the effectiveness of any intervention. The frameworks behind regulation of body weight and food intake are far from being completely understood and recent research has identified a high degree of inter-individual variability in the vulnerability to the obesogenic environment and the metabolic response to food and weight loss interventions, asserting that when it comes to obesity treatment, one size does not fit all [133]. Current efforts are focused on gathering all relevant genetic, epigenetic, phenotypic, medical and nutritional information in the aim of integrating them into multi-dimensional individualized recommendations that take into account individual preferences as well as metabolic profiles to maximize adherence. Excessive adiposity results from a pre-programmed genetic, epigenetic, metabolic and even gut microbial makeup, and influenced by environmental factors, which complicates all attempts for prevention and treatment of obesity and its associated comorbidities. Nevertheless, lifestyle modification and proper nutrition remain a crucial part of the solution to obesity and an effective approach that can greatly influence much of the modifiable risk factors. At the population level, whole system approaches (WSA) that recognize the complexity and multifaceted nature of obesity are being increasingly used to address this epidemic at the national, regional and international levels. The main features of WSA involve acknowledging the multifactorial drivers of obesity, coordinating actions between multiple stakeholders including non-healthcare related players, operating at all levels of governance and targeting all age groups [134]. A systematic review by Bagnall et al. (2019) reported a range of positive health outcomes resulting from the adoption of WSA in the context of obesity and related public health issues in multiple countries; these include improvements in BMI, awareness and health behaviors among others [134]. Such findings highlight the importance of addressing the food and built environments as essential drivers of obesity and NCDs and adopting a holistic approach that targets food systems from production to consumption in curbing down obesity and its associated morbidities. Future research should continue to explore the options to optimize dietary approaches in order to deliver more integrative, personalized and country-specific nutritional guidance.

## Figures and Tables

**Table 1:** Examples of population-wide interventions to combat obesity.

**Targeting Individual Behaviors**
Mass media campaigns
Nutrition counselling and education in healthcare settings
**Targeting Food and Built Environment**
Restaurant menu labelling
Dietary guidelines/standards
Advertisement restrictions
Interventions targeting schools and universities
Regulations/policies
Fiscal measures including taxes and subsidies

**Table 2:** Selected systematic reviews and meta-analyses comparing macronutrient diets for weight loss effectiveness.

Reference	Sample Size	Duration	Comparison	Result
Hooper et al. (2012) [49]	73,589 adults with normal weight, overweight or obesity	6 m–7.5 years	Low fat (>30%) vs. Normal Fat	Low fat diets produced greater weight loss by 1.57 kg (95% CI −2.0 to −1.2 kg)
Wycherley et al. (2012) [50]	1063 adults, BMI status not specified	>12 weeks	High protein (27–35%) vs. Low fat	High protein diets produced greater weight loss by 0.79 kg (95% CI −1.5 to −0.08 kg)
Ajala et al. (2013) [51]	3073 adults with normal weight, overweight or obesity with or without diabetes	>6 months	Low carbohydrate vs. Mediterranean diet vs. High protein	Mediterranean diet and low carbohydrate diets produced greater weight loss than high protein by 1.84 and 0.69 kg respectively
Bueno et al. (2013) [52]	1415 adults, BMI status not specified	≥12 months	Very low carbohydrate (<50 g) vs. low fat (<30%, energy restricted)	Very low carbohydrate diets achieved greater weight loss by 0.91 (95% CI −1.65 to −0.17 kg)
Naude et al. (2014) [53]	3209 adults with overweight or obesity with or without diabetes	>12 weeks	Low carbohydrate vs. isocaloric balanced diets	No difference
Clifton et al. (2014) [54]	3492 adults with overweight or obesity	>12 months	High protein (>25%) vs. usual diet	High protein diets produced greater weight loss by 1 kg
Tobias et al. (2015) [55]	68,128 adults, BMI status not specified	≥12 months	Low fat vs. usual diets, low carbohydrate, high fat	Low carbohydrate diets produced greater weight loss by 1.15 kg (95% CI −0.52 to −1.79)
Boaz et al. (2015) [56]	1161 adults with normal weight, overweight or obesity	>12 weeks	Low carbohydrate vs. Low fat	No difference
Mansoor et al. (2016) [57]	1369 adults, BMI status not specified	≥6 months	Low fat vs. low carbohydrates	Low carbohydrate diets produced greater weight loss by 2.17 kg (95% CI −0.99 to −3.36 kg)
Ge et al. (2020) [58]	21,942 adults with overweight or obesity	6–12 m	14 popular diets including low fat, low carbohydrate, Mediterranean Diet	Low carbohydrate and low-fat diets produced similar weight loss, greater than Mediterranean diet

**Table 3:** Key dietary factors with associated epigenetic metabolic effects.

Nutritional Factors	Potential Epigenetic Mechanisms	Associated Metabolic Outcomes
Caloric restriction/undernutrition	DNA methylation Histone modifications miRNA	Increased risk of obesity and metabolic disorders (including insulin resistance and diabetes) in adulthood
Fatty Acids		
Unsaturated FA (MUFA, n-3 PUFA)	DNA methylation Histone modifications miRNA	Prevention of metabolic derangements (lipid metabolism disturbances, inflammation, and insulin resistance) or chronic diseases (obesity, diabetes, non-alcoholic fatty liver disease, CVD risk and some types of cancer) (Becerra, 2019)
n-6 PUFA, Saturated and trans FA	DNA methylation Histone modifications miRNA	Associated with the presence or development of obesity, diabetes, inflammatory profile, atherosclerosis, hyperglycemia, insulin resistance, lipid alterations, lipotoxicity, dysregulation of lipid metabolism, and abnormal lipid accumulation (Becerra, 2019)
SCFA	DNA methylation Histone modifications Interaction with Gut Microbiome	Mixed effects
Methyl donors (e.g., folate, methionine, choline) *	DNA methylation	Increased risk of metabolic disorders including insulin resistance, diabetes and obesity
Probiotics/Fibers	Production of SCFA (same as SCFA)	Same as SCFA
Vitamins and minerals (e.g., retinol, ascorbate, tocopherol)	DNA methylation Histone modifications	Antioxidant effects
Polyphenols and other plant compounds	Inhibition of DNA hypermethylation Histone modification miRNA	May protect against carcinogenesis and obesity on adulthood
Dietary Patterns		
High fat diet/Western diet	DNA methylation of obesity related genes leading to changes in genes expression and appetite regulation	- Imbalance in fatty acids causing oxidative stress and inflammatory state - Increased risk of obesity and metabolic disorders (including insulin resistance and diabetes) in adulthood
Mediterranean diet	DNA methylation	Protective against CVD risk and cancer
Intermittent Fasting	Improve the expression of anti-oxidant and anti-inflammatory genes	May be protective against oxidative stress

Based on evidence from [128,129,130,131]. * Deficiency is associated with negative outcomes perinatally as well as during adulthood; negative outcomes can be reversed by supplementation of methyl donor [130].
